# Fault-Tolerant Algorithms for Connectivity Restoration in Wireless Sensor Networks

**DOI:** 10.3390/s16010003

**Published:** 2015-12-22

**Authors:** Yali Zeng, Li Xu, Zhide Chen

**Affiliations:** Fujian Provincial Key Laboratory of Network Security and Cryptology, School of Mathematics and Computer Science, Fujian Normal University, Fuzhou 350007, China; zhidechen@fjnu.edu.cn

**Keywords:** wireless sensor networks, connectivity restoration, fault tolerance

## Abstract

As wireless sensor network (WSN) is often deployed in a hostile environment, nodes in the networks are prone to large-scale failures, resulting in the network not working normally. In this case, an effective restoration scheme is needed to restore the faulty network timely. Most of existing restoration schemes consider more about the number of deployed nodes or fault tolerance alone, but fail to take into account the fact that network coverage and topology quality are also important to a network. To address this issue, we present two algorithms named Full 2-Connectivity Restoration Algorithm (F2CRA) and Partial 3-Connectivity Restoration Algorithm (P3CRA), which restore a faulty WSN in different aspects. F2CRA constructs the fan-shaped topology structure to reduce the number of deployed nodes, while P3CRA constructs the dual-ring topology structure to improve the fault tolerance of the network. F2CRA is suitable when the restoration cost is given the priority, and P3CRA is suitable when the network quality is considered first. Compared with other algorithms, these two algorithms ensure that the network has stronger fault-tolerant function, larger coverage area and better balanced load after the restoration.

## 1. Introduction

Wireless sensor networks (WSNs) are known for their wide use in industry, military, and environmental monitoring applications [1]. They are usually deployed in harsh environments, where nodes are subjected to failures and the networks are easy to be partitioned into disjoint segments. Therefore, fault tolerance becomes a critical issue for WSNs and numerous restoration algorithms are proposed [2,3,4,5,6] to address this issue. In order to achieve fault tolerance when restoring a faulty WSN, one approach is to deploy additional relay nodes to provide *k* (*k* > 1) vertex-disjoint paths (hereinafter referred to as *k*-connectivity) between every pair of network nodes (segments and relay nodes). In this way, the restored network can survive the failure of fewer than *k* nodes, which is more practical for WSNs. In this paper, we adopt this approach to repair the faulty network which is divided into many segments.

However, deploying additional relay nodes for network restoration brings us two conflicting requirements: On the one hand, it needs to spend some money to purchase the equipment. In order to save the cost, it is required to place as few nodes as possible to repair the faulty network. On the other hand, as a wireless sensor network is easy to fail, the network after the restoration is required to be with fault-tolerant function so that it can resist the attack and damage in the future. The network, which is constructed by using as few nodes as possible, may not be fault-tolerant, but the network with fault-tolerant function needs to deploy more relay nodes and costs more money. Hence, these two requirements are contradictory. In addition, as for a network, network coverage and topology quality are also important to a network. Therefore, when designing the restoration scheme, we should consider not only the cost and network fault tolerance, but also the other aspects. Only in this way can the network after the restoration be more practical.

### 1.1. Our Contributions

In this paper, we comprehensively consider the restoration cost, fault tolerance, network coverage and topology quality. We seek to use fewer nodes to establish a network with fault-tolerant function under the premise of multiple segments that are unable to communicate with each other. Meanwhile, except for the restoration cost and fault tolerance, we also consider the network coverage, the quality of topology and others in this paper, so as to ensure that the network can not only has better fault tolerance, but also has stronger robustness and higher coverage after the restoration. Certainly, these performances are not considered fully in the existing literature. The algorithms we propose in this paper are summarized as follows:

(1) Full 2-Connectivity Restoration Algorithm (F2CRA) provides two vertex-disjoint paths between every pair of network nodes. This algorithm is suitable when the cost is considered first.
(2) Partial 3-Connectivity Restoration Algorithm (P3CRA) provides three vertex-disjoint paths between every pair of segments and at least two vertex-disjoint paths between every pair of relay nodes. This algorithm is suitable when the fault tolerance, network coverage and topology quality are considered first.

### 1.2. Paper Organization

The remainder of this paper is organized as follows. Section 2 reviews some related works. Section 3 proposes the system model and preliminaries. Our algorithms are introduced in Section 4. Section 5 and Section 6 conduct the theory and simulation analysis for our algorithms, respectively. Finally, we conclude this paper in Section 7.

## 2. Related Work

WSNs are prone to failures due to the hostile environments where they are deployed. How to recover a faulty WSN is an important issue that has attracted numerous researches. We summarize some existing restoration algorithms in Table 1. In the connected relay node placement problem, the aim is to ensure the network is connected (*k* = 1) [7,8,9,10,11,12,13], while in the survivable relay node placement problem, the aim is to ensure k-connectivity (*k* > 1) [2,3,4,5,6,14,15,16,17,18]. *k*-connectivity can be either full or partial [19]. Full *k*-connectivity implies that *k* node-disjoint paths exist between every pair of nodes, while partial fault-tolerance requires *k*-connectivity between original nodes (segments) only.

**Table 1:** Relay placement algorithms.

Algorithms	*k*	Deployment Locations	Fault-Tolerance	Network Types
Lloyd [9]	*k* = 1	Unconstrained	No	Homogeneous
Li [10]	*k* = 1	Unconstrained	No	Heterogeneous
Bhattacharya [13]	*k* = 1	Constrained	No	Homogeneous
Yang [11]	*k* = 1, 2	Constrained	Full	Hierarchical
Hao [2]	*k* > 1	Unconstrained	Partial	Hierarchical
Zhang [3]	*k* = 2	Unconstrained	Full	Hierarchical
Han [4]	*k* > 1	Unconstrained	Full, Partial	Heterogeneous
Senel [5]	*k* = 2	Unconstrained	Full	Homogeneous
Our algorithms	*k* = 2, 3	Unconstrained	Full, Partial	Homogeneous

In connectivity problems, most algorithms restore a faulty network by finding the minimum spanning tree or Steiner tree. Lin and Xue [7] show that the STP-MSP problem is NP-hard. They also show that the approximation obtained from the minimum spanning tree has a worst-case performance ratio at most 5, while Chen *et al.* [8] point out that this approximation has a performance ratio exactly 4. Chen *et al.* also present a new polynomial-time approximation with a performance ratio at most 3. Yang *et al.* [11] study two-tiered constrained relay node placement problems and propose polynomial time approximation algorithms with O(1)-approximation ratios. Lloyd *et al.* [9] study two versions of relay node placement problems, but the same objective of these two versions is to deploy the minimum number of relay nodes. Li *et al.* [10] also has the same objective as [9], but they study the placement problem in a heterogeneous WSN. Although easy to implement, these algorithms are usually not efficient when a failure occurs in the network.

In survivable problems, most algorithms aim to construct a fault-tolerant network topology in a WSN. Hao and Tang *et al.* [2,14] study a fault-tolerant relay node placement problem in a two-tiered network, while Zhang *et al.* [3] study the problem in both single and two tiered networks. Smith *et al.* [2] is further extended to cover *k*-connectivity in heterogeneous wireless sensor networks in [4] where sensor nodes possess different transmission radii. The same as [4], Misra *et al.* [15] study the placement problem in heterogeneous wireless sensor networks, but [15] studies a constrained version in which relay nodes can only be placed at a set of candidate locations. As many algorithms do in connectivity problem, many restoration algorithms in survivable problem also try to place fewest number of relay nodes in a WSN like [6,16,17].

In a word, most of the aforementioned algorithms try to place minimum relay nodes in a WSN. However, none of them take network quality into account which is also crucial in terms of application-level performance. Therefore, Senel and Lee *et al.* [5,18] opt to reestablish connectivity using the least number of relays while ensuring a certain quality in the formed topology. However, their algorithms produce many overlapped areas and cannot be practical in multiple node failures caused by aftermath. To address these issues, we jointly consider establishing fault-tolerant connectivity and providing large coverage area which has not been studied.

## 3. System Model and Preliminaries

### 3.1. System Model

WSN is often deployed in the hostile environment, and sometimes it may suffer from the large-scale damage, resulting in the entire network being divided into multiple segments which cannot communicate with each other. In this paper, the problem we consider is how to repair the faulty WSN composed of multiple segments. As mentioned above, our scheme is to deploy relay nodes between each segment, but this scheme brings us two contradictory requirements: One is to minimize the number of nodes, and the other is to construct a fault-tolerant network. If the segments are regarded as a node, the set of these nodes is defined as S and the set of the deployed relay nodes is defined as P, then our problem can be transformed into the following.

Given a set of nodes (segments) S on a plane with a random distribution, the nodes in the set S cannot communicate with each other. After the set of relay nodes P being added on the plane for the restoration, all the nodes S∪P can communicate with each other. It requires that (1) the number of relay nodes is minimized; and (2) the network is fault-tolerance after the restoration.

### 3.2. Preliminaries

**Figure 1:**
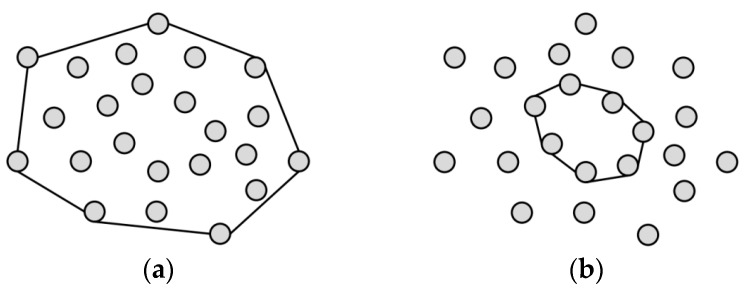
(**a**) Minimum convex hull; and (**b**) Inner convex hull.

**Definition 1.**  *Minimum Convex Hull: Given a set of nodes* S *on a plane with a random distribution, the minimal convex polygon which contains all the points is called minimum convex hull (Figure 1a). In this paper, we need to construct two convex hulls. To describe them conveniently, we call this minimum convex hull as outer convex hull here. The outer convex hull is composed of the isolated segments in the network.*

**Definition 2.**  Inner Convex Hull: In this paper, when the minimum convex hull is found, in order to ensure the network has fault tolerance after the restoration, the other convex hull is built inside the minimum convex hull. This convex hull is called inner convex hull (Figure 1b).

**Definition 3.**  Corner Point of Convex Hull: The points to make up convex hull are called corner points. In outer convex hull, corner points are the isolated segments, while in inner convex hull, corner points are relay nodes.

The notations used in this paper are as follows (Table 2).

**Table 2:** Notations.

Notation	Description
OCH	Outer Convex Hull
ICH	Inner convex hull
CP	Corner point
O	Center of OCH
R	Radius of relay node
P	Set of relay nodes
S	Set of n segments, $S=\left\{s_{1},s_{2},\ldots ,s_{n}\right\}$. The corresponding coordinate set of these n segments is $\left\{\left(x_{1},y_{1}),(x_{2},y_{2}),\ldots ,(x_{n},y_{n}\right)\right\}$.

## 4. Algorithms

This section illustrates two proposed algorithms in detail.

### 4.1. Full 2-Connectivity Restoration Algorithm

**Figure 2:**
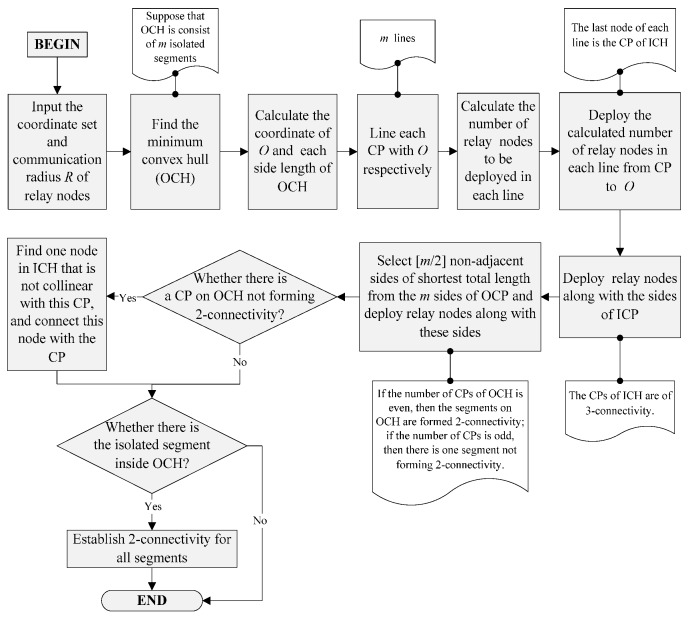
Flow chart of Full 2-Connectivity Restoration Algorithm.

Many schemes place too many relay nodes for improving network fault tolerance performance, but other performances like network coverage and topology quality have not been optimized. To address this issue, we propose a new restoration algorithm named Full 2-Connectivity Restoration Algorithm (F2CRA). This algorithm aims to deploy the minimum number of relay nodes to form a full 2-vertex connected network. Meanwhile, the restored network has a larger coverage area and a more balanced load than other schemes. The flow chart of F2CRA is shown in Figure 2.

The steps of F2CRA are as follows (Algorithms 1):

Given the scattered segments set S on the plane, in Step 1 the minimum convex hull composed of these segments is found by the method of Graham scan algorithm. The time complexity of Graham scan algorithm is O(nlogn). By calculating the length from CPs to the center of OCH, in Step 2, we obtain the number of nodes and the accurate deployment position between each CP and O. Step 2 enables the nodes on ICH to form 3-connectivity, and Steps 3 and 4 enable the remaining segments on the plane to form 2-connectivity. Steps 2–4 make the network topology have the fan-shaped structure after the restoration. Compared with other algorithms, the network topology with such structure has better fault tolerance, larger coverage and more balanced load. The time complexity from Step 2 to Step 4 is O(n); therefore, the time complexity of F2CRA algorithm is O(nlogn).

**Algorithms 1 F2CRA****INPUT:**
R, $S=\left\{s_{1},s_{2},\ldots ,s_{n}\right\}$ and $\left\{\left(x_{1},y_{1}),(x_{2},y_{2}),\ldots ,(x_{n},y_{n}\right)\right\}$. P is null. **OUTPUT:** A set of relay nodes
P.Step 1. Find OCH.  (1) Adopt the Graham scan algorithm to find OCH in
S.    Suppose that CPs set of OCH is $\left\{s_{1},s_{2},\ldots ,s_{m}\right\}\subset S$ and their corresponding coordinate is $\left\{\left(x_{1},y_{1}),(x_{2},y_{2}),\ldots ,(x_{m},y_{m}\right)\right\}$. The remaining segments set inside OCH is $\left\{s_{m+1},s_{m+2},\ldots ,s_{n}\right\}$.  (2) Calculate the coordinate $\left(x_{0},y_{0}\right)$ of O.   $x_{0}=\frac{1}{m}\sum_{i=1}^{m}x_{i}$   $y_{0}=\frac{1}{m}\sum_{i=1}^{m}y_{i}$  (3) Calculate each side length of OCH.       **for**
i=1 to m
**do**        j=i+1       **if**
i=m
**then**        j=1       **end if**       $side_{i}=\sqrt{{\left(x_{i}-x_{j}\right)}^{2}+{\left(y_{i}-y_{j}\right)}^{2}}$      **end for**   Then the set of the side lengths of OCH is $\left\{side_{1},side_{2},\ldots ,side_{m}\right\}$.Step 2. Find ICH.  (1) Line each CP with O, respectively, and calculate the length of each line:    **for**
i=1 to m
**do**    $l_{i}=\sqrt{{\left(x_{i}-x_{0}\right)}^{2}+{\left(y_{i}-y_{0}\right)}^{2}}$      Then the set of these lines is $\left\{l_{1},l_{2},\ldots ,l_{m}\right\}$  (2) Calculate the angle between two adjacent lines:    **for**
i=1 to m
**do**    j=i+1       **if**
i=m
**then**        j=1        **end if**      $\theta_{i}=\arccos\left(\frac{l_{i}^{2}+l_{i+1}^{2}-side_{i}^{2}}{2\times l_{i}\times l_{i+1}}\right)$      Calculate the number of relay nodes to be deployed in each line:    $x_{i}^{\prime }=x_{0}\pm \frac{R\times \left|x_{0}-x_{i}\right|}{2\times l_{i}\times \sin\frac{\theta_{i}}{2}}$,    $y_{i}^{\prime }=y_{0}\pm \frac{R\times \left|y_{0}-y_{i}\right|}{2\times l_{i}\times \sin\frac{\theta_{i}}{2}}$    $n_{i}^{\prime }=\left\lfloor \frac{\sqrt{{\left(x_{i}-x_{i}^{\prime }\right)}^{2}+{\left(y_{i}-y_{i}^{\prime }\right)}^{2}}}{R}\right\rfloor$    Start with a CP of OCH $s_{i}$, and deploy relay nodes towards O with one relay node every distance R, then get the corresponding deployment position of $n_{i}^{\prime }$ nodes. Add these nodes into the set of P and set the last node $p_{i}$ , the coordinate $\left(x_{i}^{\prime \prime },y_{i}^{\prime \prime }\right)$.      **end for**    Then the CPs set of ICH is $\left\{p_{1},p_{2},\ldots ,p_{m}\right\}$.  (3) Deploy the nodes along with the edge of ICH.    **for**
i=1 to m
**do**        **if**
i=m
**then**         j=1     **end if**    Deploy relay nodes between $p_{i}$ and $p_{i+1}$, and add these nodes into the set of P.  **end for**Step 3. Establish 2-connectivity for the segments on OCH.     **if**
m%2=0
**then**    Select $\frac{m}{2}$ non-adjacent sides of shortest total length from the side set $\left\{side_{1},side_{2},\ldots ,side_{m}\right\}$, deploy relay nodes along with these sides, and add these nodes into the set of P.   **else **    There will be a vertex u∈S not forming 2-connectivity. At this time, find a node v on ICH that is closest to u but not collinear with u, deploy the nodes uniformly between v and u , and add these nodes into the set of P.   **end if**Step 4. Establish 2-connectivity for the isolated segments on the plane   **for**
k=m+1 to n
**do**    Find the nearest two nodes $u^{\prime }$ and $v^{\prime }$ for $s_{k}$, $u^{\prime },v^{\prime }\in P$. Deploy relay nodes uniformly in $s_{k}$ and $u^{\prime }$, $s_{k}$ and $v^{\prime }$, and add these nodes into set P.  **end for**

### 4.2. Partial 3-Connectivity Restoration Algorithm

**Figure 3:**
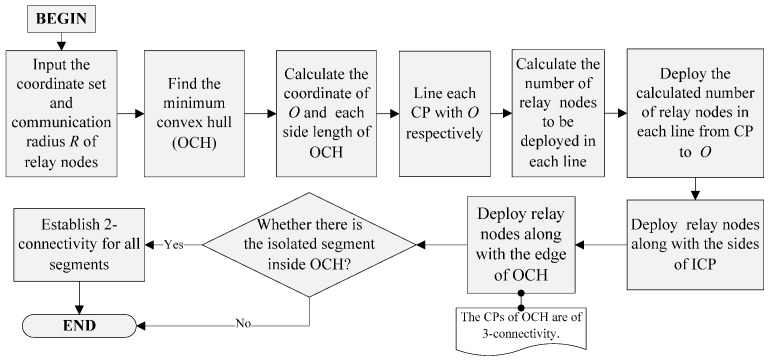
Flow chart of Partial 3-Connectivity Restoration Algorithm.

F2CRA uses fewer nodes to establish a network topology with fault tolerance. Therefore, F2CRA is suitable for the case when the number of available relay nodes is small. When the number of available nodes is sufficient, we can extend F2CRA, so that the network topology can have the stronger fault tolerance after the restoration. Here, we propose an improved algorithm Partial 3-Connectivity Restoration Algorithm (P3CRA). P3CRA is similar to F2CRA, but the network restored by P3CRA will have partial 3-connectivity structure. Partial 3-connectivity means that after the restoration, all the segments have 3-connectivity at least, and the deployed relay nodes have 2-connectivity at least. The network restored by P3CRA has larger coverage and better fault tolerance than that by F2CRA. However, P3CRA needs to deploy more nodes; therefore, P3CRA is suitable when the network quality is taken into consideration first, and F2CRA is suitable when the cost is in consideration. P3CRA flow chart is shown in Figure 3.

The steps of P3CRA are as follows (Algorithms 2):
 **Algorithms 2 P3CRA** **INPUT:**
R, $S=\left\{s_{1},s_{2},\ldots ,s_{n}\right\}$ and $\left\{\left(x_{1},y_{1}),(x_{2},y_{2}),\ldots ,(x_{n},y_{n}\right)\right\}$. P is null. **OUTPUT:** A set of relay nodes
P. Step 1 and Step 2 are the same with F2CRA’s. Step 3. Establish 3-connectivity for the segments on OCH.   **for**
i=1 to m
**do**     Deploy relay nodes between
$s_{i}$ and $s_{i+1}$.(if i=m, then i+1=1)   **end for** Step 4. Establish 3-connectivity for the isolated segments on the plane.    **for**
k=m+1 to n
**do**     Find the nearest three nodes $u^{\prime }$, $v^{\prime }$ and $w^{\prime }$for $s_{k}$. ($u^{\prime }$, $v^{\prime }$, $w^{\prime }$are not on the same line)     Deploy nodes uniformly in
$s_{k}$ and $u^{\prime }$, $s_{k}$ and $v^{\prime }$, $s_{k}$ and $w^{\prime }$.   **end for**

The first two steps are consistent in F2CRA algorithm and P3CRA algorithm, but in Step 3, P3CRA algorithm directly deploys nodes along the edge of OCH. At that time, all CPs (segments) on OCH form 3-connectivity. In Step 4, all segments on the plane eventually form 3-connectivity. Like F2CRA algorithm, the time complexity of P3CRA algorithm is also O(nlogn).

To summarize, the network topology repaired by F2CRA algorithm has 2-connectivity. As it needs fewer nodes, this algorithm is suitable when the cost is considered first. Compared with F2CRA algorithm, P3CRA algorithm needs to deploy more nodes. Due to the stronger fault tolerance, larger coverage and more balanced load, P3CRA algorithm is applicable when the performance of network is considered first.

## 5. Algorithm Analysis

It is known that the coordinates of CPs and the center coordinate of OCH are $\left\{\left(x_{1},y_{1}),(x_{2},y_{2}),\ldots ,(x_{n},y_{n}\right)\right\}$ and $\left(x_{0},y_{0}\right)$, respectively, and the value of communication radius of relay nodes is R. Assume that nodes are deployed every distance R. When the CP coordinate of ICH is ($x_{0}\pm \frac{R\times \left|x_{0}-x_{i}\right|}{2\times l_{i}\times \sin\frac{\theta_{i}}{2}}$,$y_{0}\pm \frac{R\times \left|y_{0}-y_{i}\right|}{2\times l_{i}\times \sin\frac{\theta_{i}}{2}}$), the restoration algorithm will use the minimum number of relay nodes.

**Figure 4:**
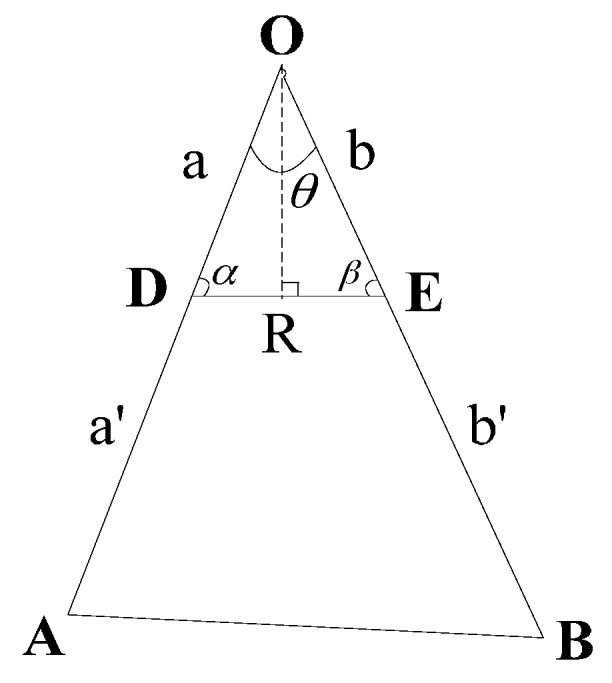
Diagram of triangle.

**Proof.**  As shown in Figure 4, we assume the coordinates of point A, B, and O are $\left(x_{1},y_{1}\right)$, $\left(x_{2},y_{2}\right)$, and $\left(x_{0},y_{0}\right)$, respectively. Here point A and point B represent the different CPs of OCH, and point O represents the center of OCH. Our algorithm deploys relay nodes from the CPs (A and B) to the central point (O). As the values of AB,AO,BO and R are fixed, to minimize the nodes, it requires the total length of AD and BE to be the shortest, that is, the total length of OD and OE is the longest. Consequently, the problem is transformed into: Seeking the coordinate values of point D and E when the total length of OD and OE is the longest.

Set ∠AOB=θ, the lengths of DE, OD and are R, a and b, respectively. ∠ODE=α, ∠OED=β, a, b, α, β are unknown. When a is equal to b, a+b reaches the maximum value, which means the total length of OD and OE is the longest. The detailed argument is relegated to the Appendix.

When a=b, by trigonometric function, we have:

(1) $$a=b=\frac{\frac{R}{2}}{\sin\frac{\theta }{2}}=\frac{R}{2\sin\frac{\theta }{2}}$$

Assume the coordinate of point D is $\left(x_{1}^{\prime },y_{1}^{\prime }\right)$, then:

(2) $$\sqrt{{\left(x_{1}^{\prime }-x_{0}\right)}^{2}+{\left(y_{1}^{\prime }-y_{0}\right)}^{2}}=\frac{R}{2\sin\frac{\theta }{2}}$$

As D is on line AO, then we have:

(3) $$\frac{y_{0}-y_{1}}{x_{0}-x_{1}}=\frac{y_{0}-y_{1}^{\prime }}{x_{0}-x_{1}^{\prime }}$$

By Equations (2) and (3), we have:

(4) $$x_{1}^{\prime }=x_{0}\pm \frac{R\times \left|x_{0}-x_{1}\right|}{2\times l_{1}\times \sin\frac{\theta }{2}}$$

(5) $$y_{1}^{\prime }=y_{0}\pm \frac{R\times \left|y_{0}-y_{1}\right|}{2\times l_{1}\times \sin\frac{\theta }{2}}$$

If $x_{1}<x_{0}$, then:

(6) $$x_{1}^{\prime }=x_{0}-\frac{R\times \left|x_{0}-x_{1}\right|}{2\times l_{1}\times \sin\frac{\theta }{2}}$$

Otherwise:

(7) $$x_{1}^{\prime }=x_{0}+\frac{R\times \left|x_{0}-x_{1}\right|}{2\times l_{1}\times \sin\frac{\theta }{2}}$$

$y_{1}^{\prime }$ is similar to $x_{1}^{\prime }$, and the method to get the coordinate of E is similar to that of D. That is, when the coordinates of D and E are, respectively, $\left(x_{0}\pm \frac{R\times \left|x_{0}-x_{1}\right|}{2\times l_{1}\times \sin\frac{\theta }{2}},y_{0}\pm \frac{R\times \left|y_{0}-y_{1}\right|}{2\times l_{1}\times \sin\frac{\theta }{2}}\right)$ and $\left(x_{0}\pm \frac{R\times \left|x_{0}-x_{2}\right|}{2\times l_{2}\times \sin\frac{\theta }{2}},y_{0}\pm \frac{R\times \left|y_{0}-y_{2}\right|}{2\times l_{2}\times \sin\frac{\theta }{2}}\right)$, the total length of OD and OE is the longest, the total length of AD and BE is the shortest, and the number of nodes is the least.

To summarize, the number of the nodes can be the least when the CP coordinate of ICH is

$$\left(x_{0}\pm \frac{R\times \left|x_{0}-x_{i}\right|}{2\times l_{i}\times \sin\frac{\theta_{i}}{2}},y_{0}\pm \frac{R\times \left|y_{0}-y_{i}\right|}{2\times l_{i}\times \sin\frac{\theta_{i}}{2}}\right)$$.

## 6. Algorithm Comparison and Simulation Analysis

### 6.1. Algorithm Comparison

**Figure 5:**
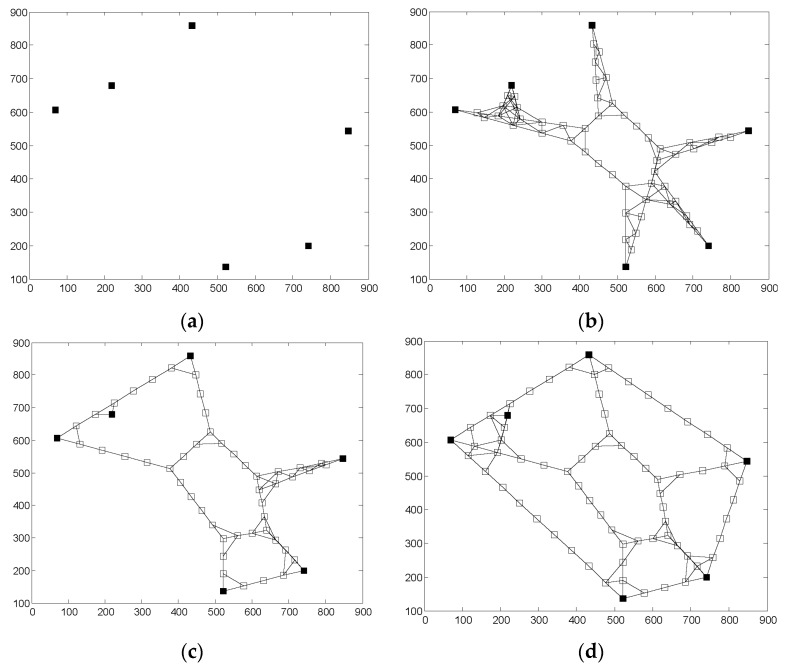
(**a**) The distribution of segments before the restoration; (**b**) 2C-SpiderWeb; (**c**) F2CRA; and (**d**) P3CRA.

Figure 5a is the distribution of segments before the restoration, where there is no mutual communication between the isolated segments. Figure 5b is the diagram of network topology structure which is restored by 2C-SpiderWeb algorithm. From Figure 5b, we can see that this network topology has a large overlapping coverage. Because of this, the network has high average degree after the restoration. However, in this case, high average degree do not represent the network has better fault tolerance. If the localized fault occurs near the CP of OCH, such as the fire, the network restored by 2C-SpiderWeb may be divided into several segments again with high probability. Therefore, although the network has 2-connectivity after the restoration by 2C-SpiderWeb, it does not have good fault tolerance.

Figure 5c,d are, respectively, the graphs of network topology structure of F2CRA and P3CRA after the restoration. From Figure 5c, we can see that all the network nodes after the restoration have at least 2-connevtivity. With such topology structure, the network can continue to run stably when one node fails. From Figure 4d, we can see that the network topology structure formed by P3CRA has larger coverage and better fault tolerance than others’. When the localized fault occurs, both F2CRA and P3CRA can maintain the stability of the network, and the network is not easy to be divided into many segments again.

### 6.2. Simulation Analysis

**Figure 6:**
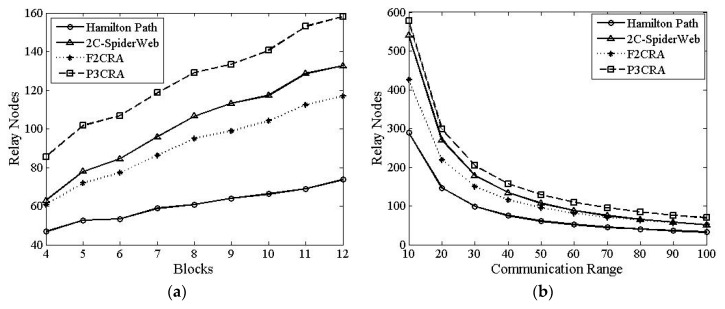
(**a**) Relay Nodes *vs.* Segments; and (**b**) Relay Nodes *vs.* Communication Radius.

**Figure 7:**
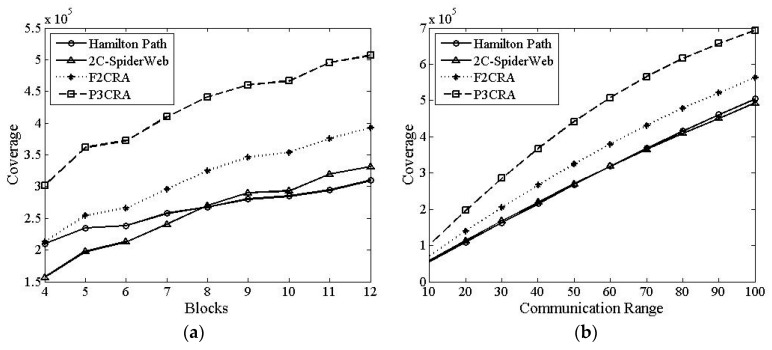
(**a**) Coverage Area *vs.* Segments; and (**b**) Coverage Area *vs.* Communication Radius.

**Figure 8:**
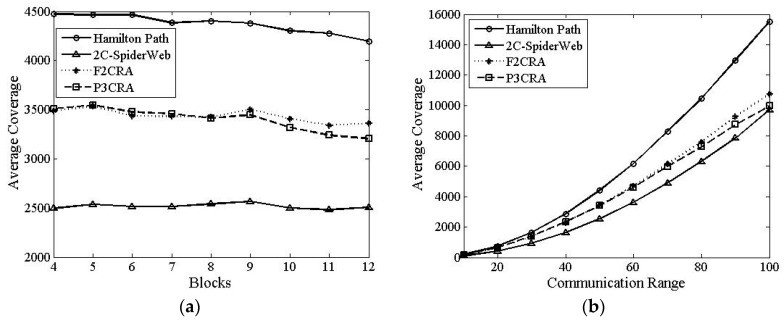
(**a**) The Average Coverage of Each Node *vs.* Segments; and (**b**) The Average Coverage of Each Node *vs.* Communication Radius.

**Figure 9:**
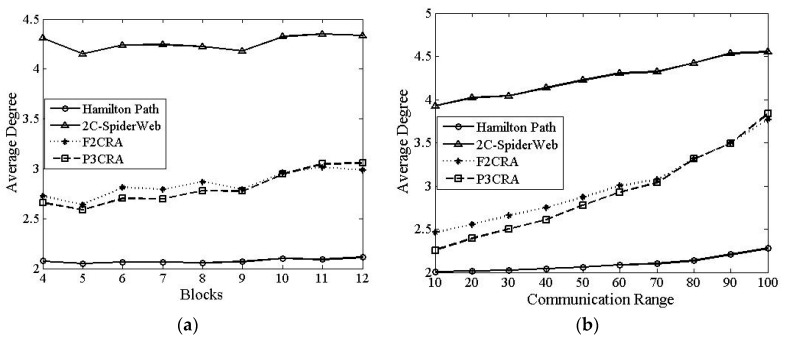
(**a**) Average Degree *vs.* Segments; and (**b**) Average Degree *vs.* Communication Radius.

In this part, we will make a comparison among Hamilton Path algorithm, 2C-SpiderWeb algorithm, F2CRA and P3CRA from the four aspects: the number of nodes (Figure 6), total coverage of nodes (Figure 7), average coverage of each node (Figure 8) and average degree (Figure 9), so as to verify the feasibility and superiority of the proposed algorithm. In this simulation, the segments are distributed on the 2D plane of 1000 × 1000 m^2^ randomly. Besides, the node communication range in Figure 6a, Figure 7a, Figure 8a and Figure 9a is fixed with the value of 50 m, and the number of segments in Figure 6b, Figure 7b, Figure 8b and Figure 9b is fixed at 8.

#### 6.2.1. The Number of Relay Nodes

From Figure 6a, we can see that when the communication radius of nodes is fixed, the number of nodes used in these four algorithms will increase with the growth of the number of segments. The more segments and the longer the total path length of the segments are, the more nodes that need to be deployed; therefore, more nodes will be used totally. It can be seen from the figure that no matter how many segments the network being divided, the nodes used in F2CRA is fewer than those in 2C-SpiderWeb algorithm, but more than those in Hamilton Path algorithm. This is determined by different topology structure of various algorithms. As P3CRA has partial 3-connectivity, the number of nodes used in this algorithm is higher than that in the other three algorithms.

From Figure 6b, we can see that the number of segments being fixed, the relay nodes used in these four algorithms are reduced with the increase of the communication radius. This is because the number of nodes is determined by the communication range of nodes when the position of segments and the distance between the segments are fixed. When the node radius is enlarged, the number of nodes between the segments is less and then the total number of nodes will be less. From Figure 6b, we can see that no matter how the radius of nodes changes, the number of nodes in P3CRA is larger than that of the other three algorithms. In addition, with the increase of the node radius, the number of nodes used in F2CRA will be more close to that in 2C-SpiderWeb algorithm. This is because the network topology formed by 2C-SpiderWeb algorithm is more similar to the one formed by F2CRA when the node radius is enlarged. As a result, the number of nodes used in these two algorithms is close.

#### 6.2.2. Total Coverage

From Figure 7, we can see that with the increase of the number of segments and the communication radius of nodes, the total coverage of these four algorithms increases. The coverage area of F2CRA is larger than that of 2C-SpiderWeb algorithm and Hamilton Path algorithm, while the coverage area of P3CRA is much larger than other three algorithms’. Although 2C-SpiderWeb algorithm has more nodes than F2CRA, the coverage area of F2CRA is always larger than that of 2C-SpiderWeb algorithm, no matter how the number of segments or the communication radius of nodes changes. From Figure 5, we can know that compared with 2C-SpiderWeb algorithm, F2CRA has a smaller overlapping area. Therefore, no matter how the number of segments or the communication radius of nodes changes, F2CRA has larger coverage area than 2C-SpiderWeb algorithm. Similarly, P3CRA uses more nodes and has smaller coverage area than F2CRA. Hence, no matter how the number of segments or the communication radius of nodes changes, P3CRA has larger coverage area than other algorithms.

#### 6.2.3. Average Coverage

From Figure 8a, we can see that the average coverage of F2CRA is basically the same with that of P3CRA, less than that of each node by Hamilton Path algorithm, but more than that of 2C-SpiderWeb algorithm. From Figure 5, we can visually know that 2C-SpiderWeb algorithm has a large overlapping area. Because of the 

large overlapping area, the actual coverage area of network topology formed by 2C-SpiderWeb algorithm becomes small. As a result, the average coverage area of each node becomes small. Compared with other three algorithms, the network topology formed by Hamilton Path algorithm has the smallest network coverage area. Consequently, the average coverage area of each node of Hamilton Path algorithm is the largest.

From Figure 8b, we can see that the average coverage area of each node will increase with the growth of the communication radius. When the position of segments is fixed, the nodes deployed between the segments depend on the communication radius of nodes. The larger the communication radius is, the smaller the number of nodes will be, but the larger the communication radius is, the larger the coverage area will be, so is the average coverage of all the nodes.

#### 6.2.4. Average Degree

From Figure 9a, we can see that the average degrees of both F2CRA and P3CR are lower than that of 2C-SpiderWeb algorithm because 2C-SpiderWeb algorithm has larger coverage overlap, and the nodes in the overlapping part have larger degree. Therefore, the average degree of this algorithm is greater than that of the other three algorithms. Moreover, the topology of Hamilton Path algorithm can be regarded as a ring, where the degree of the node is nearly 2. As some nodes overlap between them, the final average degree is slightly larger than 2. The average degrees of the two algorithms we proposed are between 2C-SpiderWeb algorithm’s and Hamilton Path algorithm’s, the reason of which can be seen clearly from the algorithm topology diagram. Compared with 2C-SpiderWeb algorithm, the two algorithms we proposed have a smaller overlapping coverage; while compared with Hamilton Path algorithm, they have a larger overlapping coverage. Hence, the final average degrees of the two algorithms we proposed are between 2C-SpiderWeb algorithm’s and Hamilton Path algorithm’s. Moreover, from Figure 9a, we can see that the average degree of P3CRA is slightly lower than that of F2CRA. The reason is that compared with F2CRA, the network topology formed by P3CRA has the smaller overlapping area; therefore, the average degree of P3CRA is slightly lower than that of F2CRA.

The case in Figure 9b is similar with that in Figure 8a, but from Figure 9b, we can see that the average degree increases with the increase of the communication radius. When the deployment location of the node is determined, the larger the communication radius of the node is, the larger the overlapping area between the nodes will be. As a result, the average degree will be larger.

## 7. Conclusions

Due to the deployment environment, WSN is prone to large-scale failure; therefore, the effective algorithm is needed for timely recovery so that the network can run normally and stably. In this paper, we propose two fault restoration algorithms, respectively, solving the WSN fault restoration problem from different points. F2CRA is suitable when cost is considered first; and P3CRA is suitable when the performances of the network are considered first. Compared with other algorithms, these two algorithms ensure that the network has the stronger fault-tolerant function, larger coverage area and more balanced load after the restoration. In future work, we plan to consider other factors of deployment environment in our algorithms, such as obstacles and rough terrain, so that the proposed algorithms can be more in line with the actual situation.

## Figures and Tables

**Figure A1:**
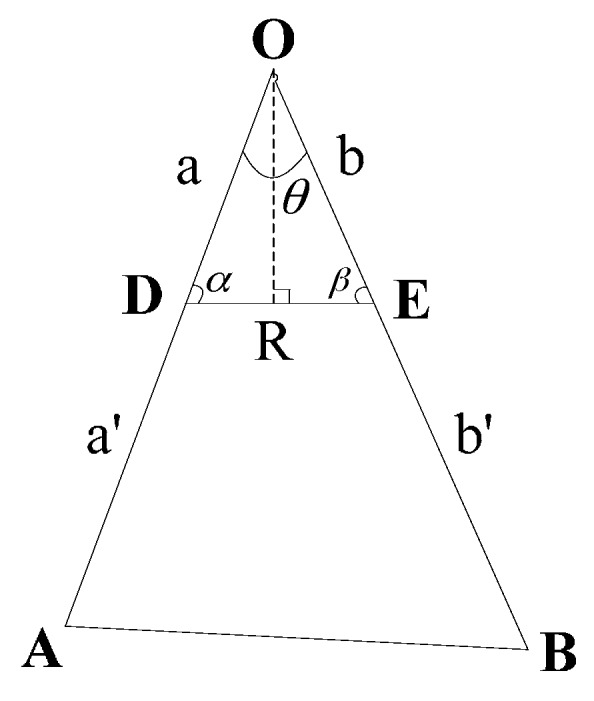
Diagram of triangle.

## Appendix

Proof of “When a is equal to b, a+b reaches the maximum value, which means the total length of OD and OE is the longest”.

As shown in Figure A1, we assume the coordinates of point A, B, and O are $\left(x_{1},y_{1}\right)$, $\left(x_{2},y_{2}\right)$, and $\left(x_{0},y_{0}\right)$, respectively. Set ∠AOB=θ, the lengths of DE, OD and OE are R, a and b, respectively. ∠ODE=α, ∠OED=β, a, α, β are unknown.

From the known, we know AB, AO and BO are, respectively:

(A1) $$side_{1}=\sqrt{{\left(x_{1}-x_{2}\right)}^{2}+{\left(y_{1}-y_{2}\right)}^{2}}$$

(A2) $$l_{1}=\sqrt{{\left(x_{1}-x_{0}\right)}^{2}+{\left(y_{1}-y_{0}\right)}^{2}}$$

(A3) $$l_{2}=\sqrt{{\left(x_{2}-x_{0}\right)}^{2}+{\left(y_{2}-y_{0}\right)}^{2}}$$

From Equations (A1)–(A3) and the cosine law, we can obtain the value of θ:

(A4) $$\theta =\arccos\left(\frac{l_{1}^{2}+l_{2}^{2}-side_{1}^{2}}{2\times l_{1}\times l_{2}}\right)$$

By trigonometric function, we have:

(A5) $$\frac{R}{\sin\theta }=\frac{a}{\sin\alpha }=\frac{b}{\sin\beta }=\frac{a+b}{\sin\alpha +\sin\beta }$$

So

(A6) $$a+b=\frac{\sin\alpha +\sin\beta }{\sin\theta }R\begin{array}{l} =\frac{2\sin\frac{\alpha +\beta }{2}\cos\frac{\alpha -\beta }{2}}{\sin\theta }R\\ =\frac{2\sin\frac{\pi -\theta }{2}\cos\frac{\alpha -\beta }{2}}{\sin\theta }R\\ =\frac{2\cos\frac{\theta }{2}\cos\frac{\alpha -\beta }{2}}{\sin\theta }R\\ =\frac{\cos\frac{\alpha -\beta }{2}}{\sin\frac{\theta }{2}}R \end{array}$$

As θ and R are known, when α=β, $\cos\frac{\alpha -\beta }{2}$ has the maximum value, then a+b reaches the maximum value. That is, when a=b, a+b reaches the maximum value, which means the total length of OD and OE is the longest.
